# Trigger medications and patient-related risk factors for Parkinson disease psychosis requiring anti-psychotic drugs: a retrospective cohort study

**DOI:** 10.1186/1471-2377-13-145

**Published:** 2013-10-12

**Authors:** Hideyuki Sawada, Tomoko Oeda, Kenji Yamamoto, Atsushi Umemura, Satoshi Tomita, Ryutaro Hayashi, Masayuki Kohsaka, Takashi Kawamura

**Affiliations:** 1 Clinical Research Center, 8 Ondoyamacho, Narutaki, Ukyoku Kyoto 616-8255, Japan; 2 Department of Neurology, National Regional Center for Neurological Disorders and Utano National Hospital, 8 Ondoyamacho, Narutaki, Ukyoku Kyoto 616-8255, Japan; 3 Kyoto University Health Service, Kyoto, Japan

**Keywords:** Dopa, Dopamine agonist, Anticholinergic, Case-crossover, Retrospective cohort study

## Abstract

**Background:**

Psychoses such as hallucinations are a frequent non-motor problem in patients with Parkinson disease (PD) and serious psychosis requires anti-psychotic medications that worsen Parkinsonism. Although psychosis could be associated with patient-related or biological factors such as cognition, age, and severity of PD, it can also be associated with medications.

Therefore we aimed to investigate patient-related and medication-related risks of psychosis requiring anti-psychotic medications (serious psychosis).

**Methods:**

A retrospective cohort of 331 PD patients was followed for 2 years. Patient-related factors associated with risk of psychosis were identified by a survival time analysis. In patients who developed psychosis, medications during the hazard period (1-14 days before psychosis) were contrasted with those during the control periods (1 and 3 months before psychosis) using a case–crossover analysis to identify medication-related risks of psychosis.

**Results:**

Serious psychosis was detected in 52 patients and the incidence was estimated to be 116 (95% confidence interval [CI], 85-148) per 1,000 person-years. Analyses of baseline characteristics revealed the risk to be higher in patients with a modified Hoehn–Yahr stage of ≥4 (hazard ratio [HR], 2.22; 95% CI, 1.11-4.40), those with a longer duration of PD (HR, 1.25; 95% CI, 1.00-1.55, per 5 years) and those with Mini-Mental State Examination scores of ≤24 (HR, 2.66; 95% CI, 1.37-5.16). The case-crossover analysis revealed that anti-cholinergics use (HR, 19.7; 95% CI, 2.39-162) elevated the risk, while donepezil use reduced it (HR, 0.48; 95% CI, 0.27-0.85).

**Conclusions:**

Risk of psychosis was elevated by increasing severity of PD, cognitive dysfunction and duration of the disease. It was elevated by use of anti-cholinergic drugs and reduced by use of donepezil. The medication-related risk was higher in patients aged ≥ 70 years. In contrast, there was no significant medication-related risk in younger patients, suggesting different pathomechanisms between young and old patients.

## Background

Psychosis is often seen in the disease course of Parkinson disease (PD) patients receiving long-term treatment, even in those without dementia [1]. It varies from mild and transient hallucinations such as “passage hallucination” to sustained delusions [2,3]. The prevalence, including mild psychosis with preserved insight, is ~40-60% [2], so psychosis is one of the most prevalent and important non-motor complications. Although mild, simple visual hallucinations with retained insight do not require additional medical treatment, severe psychosis requires hospitalization and is associated with mortality [4]. Although visual hallucinations are associated with PD brain pathology [5], they could be associated with dopaminergic replacement therapy [6], especially that involving dopamine agonists [7-9]. Antipsychotic drugs except for clozapine [10] improve psychosis insufficiently [11,12], but worsen motor symptoms [13]. Therefore, finding out a way to prevent psychosis during treatment for PD is important. Although there is no association between hallucinations and dopamine receptor genotype or apolipoprotein ϵ genotype [14], patient-specific factors may be involved because psychosis often reoccurs in some patients and seldom occurs in others [15]. Psychosis is associated with cognitive dysfunction [15-17], which is related to non-dopaminergic neuronal degeneration [18]. Psychosis could be a composite phenomenon of patient-related or biological factors such as cognition, age, severity of PD and “trigger medications”. The purpose of the present study was to investigate the incidence of psychosis and evaluate patient-related and medication-related risk factors. To identify what medications could trigger psychosis we adopted a case-crossover design that enabled us to study the effects of transient triggers on the risk of acute events, such as psychosis [19].

## Methods

### Study design

We conducted a retrospective cohort study to investigate the associations between patient-related factors and psychosis, and then analyzed the risk associated with different medications. First, a survival time analysis was adopted to identify patient-related factors. Then, with adjustment for these factors, a case-crossover design study (self-matched comparison that compared hazard and control periods in each patient with psychosis) was performed to evaluate the risk associated with medications.

This study was approved by the Bioethics Committee of Utano National Hospital (No.18-16). According to the Bioethics Committee, informed patient consent was not needed because the study was a retrospective review and data were analyzed anonymously.

### Patients

The medical records of consecutive patients with PD who were treated in the Department of Neurology, the National Regional Center for Neurological Disorders and Utano National Hospital from March 2004 to November 2007 were retrospectively reviewed over a period of two years. Inclusion criteria were as follows. Patients who were diagnosed with Parkinson disease according to the United Kingdom Parkinson’s disease Brain Bank Diagnostic Criteria (step 1 and 2) [20], and who were prescribed dopaminergic replacement therapy. Irrespective of age, sex and severity of PD, both outpatients and hospitalized patients were included. Patients with a past history of psychosis were also included. Exclusion criteria were as follows; patients prescribed anti-psychotic drugs during the preceding 1 month; patients with a history of schizophrenia; and patients who met the DLB consensus criteria for probable or possible dementia with Lewy bodies [21].

### Definition of psychosis

The primary study outcome was serious psychoses, defined as those requiring a prescription of anti-psychotic drugs. According to the guidelines of PD psychosis, we assumed that prescription of anti-psychotic drugs was justified principally when psychosis was not improved by other treatments [22,23] and patient suffering and behavioral changes were severe enough to endanger the patients or others. The diagnosis of psychosis was made based on the presence of psychotic symptoms (illusions, false sense of presence, hallucinations, or delusions) that were recurrent or prolonged for ≥1 month according to the provisional diagnostic criteria for PD psychosis [24]. Anti-psychotic drugs included all anti-psychotic drugs available in Japan; tiapride, sulpiride, risperidone, chlorpromazine, thioridazine, fluphenazine, propericiazine, levomepromazine, haloperidol, quetiapine, olanzapine, perospirone, and aripiprazole. Prescriptions were collected longitudinally from study enrollment to the endpoint, which was defined as the occurrence of psychosis that required anti-psychotic drugs or the end of the 730 days of the study period.

### Survival time analysis

In a survival time analysis, the observation period, censoring, and endpoint were defined as follows. The observation period was from the time of study enrollment to the endpoint, which was defined as the occurrence of any psychosis that required anti-psychotic drugs or the end of the 730-day study period. Observation was censored if patients were *lost to follow-up* or experienced *an alternative outcome*. Patients were censored when they were transferred to other hospitals because data could not be obtained (*lost to follow-up*). Because anti-psychotic medications were often prescribed prophylactically by surgeons to avoid surgery-associated delirium, patients who underwent surgery were also censored just before surgery (*alternative outcome*).

In patients with motor fluctuations modified Hoehn-Yahr (mH-Y) stage was evaluated in “ON period.” Although age and duration of PD increased during the study period, and mH–Y stage and Mini-Mental State Examination (MMSE) scores might deteriorate, these variables were collected at the time of enrollment on the assumption that changes in them would not have much influence on the results of the survival time analysis.

### Case-crossover analysis

For the case-crossover analysis, the prescriptions in the hazard period and those in the control periods were compared. Medications were prescribed every 14 days or 28 days in most cases, and drugs that were taken 1 day before the endpoint (the start of anti-psychotic medications) had been taken for 14 or 28 days before the endpoint; therefore, medications taken 1 day before the endpoint represent those taken for 14 days before the endpoint. In this context, the prescription 1 day before the endpoint was regarded as that in the hazard period. Medications in the hazard period were compared with those in the control periods (30 and 90 days before psychosis) (Additional file 1: Figure S1).

Prescription information was collected consecutively because it was changeable weekly or monthly. All prescriptions were collected to investigate the prescription of antipsychotic drugs throughout the observation period (of up to 2 years). Patients who had never been prescribed antipsychotic drugs within the observation period were regarded as controls or censored. In cases where antipsychotic drugs were prescribed, the reason why the antipsychotic drugs were prescribed was confirmed based on medical records.

To identify trigger medications, the doses of dopaminergic agents including L-Dopa, entacapone, dopamine agonists, amantadine and selegiline were collected. L-Dopa dose was calculated using the following formula: 1.0 × regular levodopa dose *or* 1.25 × regular levodopa dose if taking entacapone. Dopamine agonist dose was calculated as the L-Dopa equivalent dose (LDED) according to the following formula [17,25]:

$$\begin{array}{l} \mathrm{LDED}\left(\mathrm{mg}\right)=\mathrm{pramipexole}\left(\mathrm{mg}\right)\times 67+\mathrm{ropinirole}\left(\mathrm{mg}\right)\\ \;\;\;\times 25+\mathrm{pergolide}\left(\mathrm{\mu g}\right)\times 67+\mathrm{cabergoline}\left(\mathrm{mg}\right)\\ \;\;\;\times 67+\mathrm{bromocriptine}\left(\mathrm{mg}\right)\times 10\\ \;\;\;+\mathrm{talipexole}\left(\mathrm{mg}\right)\times 67 \end{array}$$

Because psychosis could be related to anticholinergic drugs, records of prescription of central anticholinergic drugs (trihexyphenidyl, piroheptine, biperiden, and promethazine) and that of donepezil (a central cholinergic drug) were collected. Records of prescription of rivastigmine were not collected because this drug was unavailable in Japan during the study period.

### Statistical analysis

The incidence of psychosis was estimated as the number of patients with psychosis divided by the corresponding person-years at risk. The survival time was defined as the duration from enrollment to the first occurrence of psychosis. The risk of psychosis owing to patient-related factors was estimated using the Cox proportional hazard model incorporating age, sex, duration of PD, mH–Y stage (1.0-3.0 *versus* 4-5), and MMSE scores (≤ 24 *versus* > 24) at study enrollment as predictable variables. Age and sex were entered into the model and the other factors were selected according to a backward stepwise likelihood ratio test (**Analysis I**). Considering the patient-related factors identified in **Analysis I**, medications were analyzed in a case-crossover study design. Doses of L-Dopa, dopamine agonists, amantadine, and selegiline, as well as the use of anticholinergic drugs, were analyzed. Donepezil hydrochloride, an inhibitor of brain acetylcholine esterase, was also included in the analysis. The relative risk was then estimated using a generalized estimating equation that is the most appropriate technique for case-crossover designs [26-28]. Link function was logistic and an autoregressive working correlation matrix was adopted because the data were collected longitudinally and it was assumed that the correlation of medication dose depends on the intervals between data collection time points. A variance-covariance matrix was estimated using the Huber-White sandwich estimator. All two-way interactions between predictable factors or covariates were examined and interactions were considered and they were incorporated in the analysis if statistically significant (**Analysis II**). Because risk of psychosis owing to medications may be enhanced in elderly patients, the case-crossover analysis was also performed in the subgroup of patients aged ≥ 70 years.

*P* values of less than 0.05 were considered statistically significant. Statistical analyses were performed using the statistical software program IBM SPSS version 21.

### Variables

In the multiple variable analysis patients with missing variables were excluded. Dose of L-Dopa with adjustment for entacapone and dopamine agonists, selegiline and amantadine were regarded as scale variables. Anticholinergic drugs and donepezil hydrochloride were regarded as dichotomous (use *versus* no use).

### Study size

On the basis of previous reports, incidence of psychosis was estimated 80 per 1,000 person-years 18]. The sample size was calculated to be 375 because 60 psychosis events were required within the 2-year period (multiple variable logistic analysis of six kinds of medications).

## Results

Among 417 patients with PD, 86 patients were excluded because of use of antipsychotic drugs at study enrollment; therefore, 331 patients were evaluated.

### Analysis I

**Figure 1:**
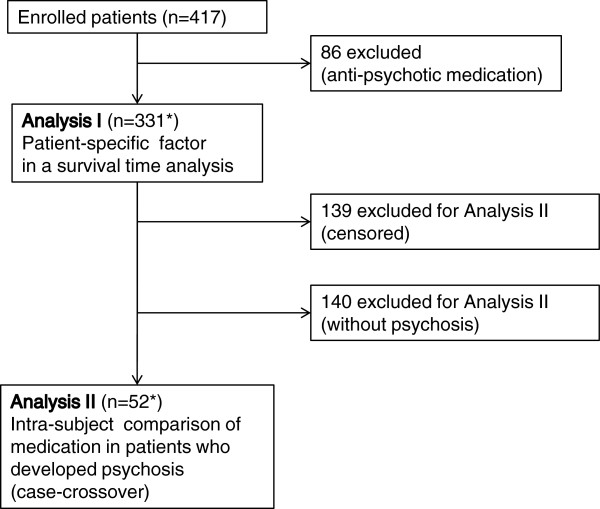
**Flow diagram of patients included in and excluded from the study.** The numbers of patients included and excluded in the analyses. The study involved **Analysis I** (patient-related factor in a survival time analysis and **Analysis II** (intra-subject comparison of medication). ^*^According to the purpose of analyses, all participants were included in **Analysis I**, and participants without psychosis were excluded from **Analysis II.**

One-hundred and ninety-two patients were followed until the occurrence of serious psychosis or the end of the study, and all prescriptions were collected longitudinally. During the study period 139 patients were censored because they were transferred to local hospitals (n = 128) or underwent surgery (n = 11). Fifty-two patients required anti-psychotic medications against psychosis (33 with visual hallucinations, 3 with auditory hallucinations, 1 with somatosensory hallucinations, and 15 with delusions). The assumption that anti-psychotic drugs were prescribed when psychotic symptoms were not improved by other treatments and the patients suffered was confirmed. No patients were prescribed anti-psychotic drugs for delirium. Two-hundred and seventy-nine patients (including the 139 that were censored) did not require them during the 2-year period (Figure 1). The incidence of psychosis was 116 (95% confidence interval [CI], 85-148) per 1,000 person-years.

**Figure 2:**
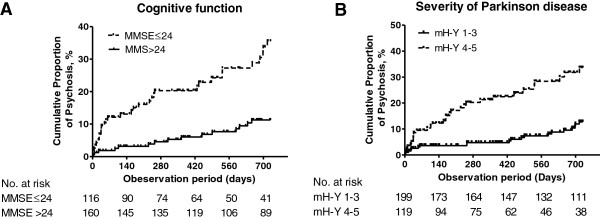
**Kaplan-Meier curves of psychosis by the severity of PD and by cognitive function.** mH–Y; modified Hoehn–Yahr stage, MMSE; Mini-Mental State Examination. **A**. Patients with MMSE scores >24 (n = 162) and those with MMSE scores ≤24 (n = 116) were compared after exclusion of 56 patients because of missing values. **B**. Patients with a mH-Y stage of 1-3 (n = 201) and those with a mH-Y stage of 4-5 (n = 120) were compared after exclusion of 13 patients because of missing values.

Characteristics of patients with and without psychosis are summarized in Table 1. Sex, mH–Y stage, MMSE score and duration of PD were significantly different between patients with psychosis and those without psychosis. Kaplan–Meier curves for the cumulative incidence of psychosis according to mH–Y stage (1-3 or 4-5), and MMSE score (≤24 or >24) are shown in Figure 2. Within the 2 years, >35% of the patients with an MMSE score ≤24 experienced psychosis, whereas only ~10% of the patients with an MMSE score >24 succumbed. More than 30% of the patients with a mH–Y stage of 4-5 developed psychosis during the study period, whereas only ~10% of the patients with a mH–Y stage of 1-3 developed psychosis. Cox hazard models demonstrated that duration of PD, mH–Y stage and MMSE score were statistically significant risk factors with age-and sex-adjusted hazard ratios (HRs) of 1.43 (95% CI, 1.17-1.73) (per 5 years), 3.39 (95% CI, 1.88-6.11) (4-5 *versus* 1-3), and 3.60 (95% CI, 1.90-6.84) (≤ 24 *versus* > 24), respectively. The multivariable-adjusted HRs were 1.25 (95% CI, 1.00-1.55), 2.22 (95% CI, 1.11-4.40) and 2.66 (95% CI, 1.37-5.16), respectively.

**Table 1:** Baseline characteristics by occurrence of psychosis

		**Psychosis (+)**	**Psychosis (-)**	**Censored**	P value***
**Characteristics**		**(n = 52)**	**(n = 140)**	**(n = 139)**	
Male, n (%)		27(51.9)	47(33.6)	68(48.9)	0.02
Age (Y), mean (SD)		70.7(6.9)	68.9(9.0)	69.4(10.2)	0.14
mH-Y stage*, n (%)	1-3	20(39.2)	107(76.4)	72(56.7)	<0.0001
	4-5	31(60.8)	33(23.6)	55(43.3)	
MMSE**	Mean (SD)	21.8(5.1)	25.6(4.2)	24.3(5.3)	<0.0001
	≤24 , n (%)	32(68.1)	37(29.8)	47(44.8)	<0.0001
	>24, n (%)	15(31.9)	87(70.2)	58(55.2)	
Age of PD onset, mean (SD)		60.5(9.9)	61.7(10.9)	61.8(12.2)	0.51
Duration (Y), mean (SD)		10.2(7.0)	7.3(5.3)	7.2(5.3)	0.002

*Abbreviations:* n, number; Y, years; SD, standard deviation; mH-Y, modified Hoehn-Yahr stage; MMSE, Mini-Mental State Examination; PD, Parkinson’s disease.

*, ** the variable totals not equal to total numbers due to missing values.

*n = 51 (psychosis (+)), 140 (psychosis (-)), 127 (censored).

**n = 47 (psychosis (+)), 124 (psychosis (-)), 105 (censored).

***comparison between pychosis (+) and psychosis (-).

### Analysis II

The correlation of medication doses among data collection time points depends on the length of the intervals (Additional file 2: Figure S2). Therefore a generalized estimating equation was adopted with an autoregressive matrix. Incorporating medications as predictable variables, the risk owing to drugs was analyzed in patients with psychosis adjusting for age, sex, duration of PD, mH–Y stage, and MMSE score. Because the interactions between anticholinergic drugs and duration of PD (HR 0.76, 95% CI, 0.64-0.89, *p* = 0.001), were highly significant, the interaction was also incorporated into the equation. There were no other significant interactions between factors. The HR for the use of anticholinergic drugs was 19.7 (95% CI, 2.39-163; *p* = 0.006). Donepezil was negatively associated with psychosis (HR 0.48; 95% CI, 0.27-0.85; *p* = 0.012) (Table 2). In the subgroup analysis of patients aged ≥70 years, use of anticholinergic drugs was positively associated with psychosis (HR 188, 95% CI, 13.9-2551, P < 0.001). Donepezil was again negatively associated with psychosis (HR 0.28, 95% CI, 0.08-1.01), and the association was statistically marginal but not significant (p = 0.051). In addition the dose of dopamine agonists was significantly associated with psychosis (HR 1.65, 95% CI, 1.02-2.66, p = 0.035). The interaction between anti-cholinergic drugs and PD duration was also significant (HR 0.62, 95% CI 0.50-0.77, p < 0.001). In contrast there were no significant associations between medications and psychosis in the subgroup of patients aged <70 years.

**Table 2:** Psychosis risk by medications (intrasubject comparison)

			**Total**		**Age <70y**		**Age > = 70y**	
			Adjusted HR* (95% CI)	***P *** **Value**	Adjusted HR* (95% CI)	***P *** **Value**	Adjusted HR* (95% CI)	***P *** **Value**
Drugs	L-Dopa	Per 100 mg /day	0.92(0.79-1.07)	0.27	1.06(0.85-1.32)	0.59	0.82(0.67-1.00)	0.05
	DA agonists	Per 100 mg(LDED) /day	1.25(0.88-1.77)	0.20	0.85(0.51-1.42)	0.53	1.65(1.02-2.66)	0.035
	Amantadine	Per 50 mg /day	0.87(0.75-1.02)	0.08	0.76(0.55-1.05)	0.09	0.86(0.63-1.17)	0.33
	Selegiline	Per 2.5 mg /day	1.06(0.85-1.33)	0.590	0.88(0.64-1.21)	0.42	1.24(0.76-2.01)	0.39
	Anti-cholinergics	Use	19.7(2.39-163)	0.006	0.80(0.38-1.68)	0.55	188.4(13.91-2551)	<0.0001
		Not use (Ref)	1		1		1	
	Donepezil use	Use	0.48(0.27-0.85)	0.012	0.79(0.51-1.22)	0.29	0.28(0.08-1.01)	0.051
		Not use (Ref)	1		1		1	
Interaction	Anti-cholinergics X duration	0.76	(0.64-0.89)	0.001			0.62(0.50-0.77)	<0.0001

Dopa were expressed as adjusted values including concomitant entacapone.

*HR was adjusted for age, sex, mH-Y (1-3 vs 4-5), MMSE (= < 24 vs >24) and duration of PD, using a generalized estimating equation.

HR was calculated for Dopa (100 mg/d, adjusted), DA agonist (LDED, 100 mg/d), selegiline (2.5 mg/d), amantadine (50 mg/d), anti-cholinergics (use vs not use), and donepezil (use vs not use).

Hazard period: prescription at psychosis occurrence.

Control period: prescription 1 month and 3 months before psychosis occurrence.

## Discussion

In this study, psychosis developed in 52 patients and was associated with disease severity, cognitive dysfunction, and longer disease duration. Furthermore, the occurrence of psychosis was associated with the use of anti-cholinergic drugs, suggesting that cholinergic neuronal degeneration could be associated with the occurrence of psychosis.

Although anti-psychotic drugs are contraindicated in PD patients, guidelines suggest that they could be used to treat psychosis when it is not improved by other treatments [22,23]. In this study, prescription of anti-psychotic drugs in such patients was justified by the reason described above. Antipsychotic drugs might also be used against agitation or aggression. However, in this study, no patients were agitated or aggressive without having delusions or hallucinations. If the occurrence of psychosis is defined solely on the basis of the use of anti-psychotic agents, mild psychosis could be overlooked. However, such a definition is a suitable method because the purpose of the study was to determine how to avoid serious psychosis requiring anti-psychotic medications. In a prospective population-based study, the incidence of psychosis was reported to be 79.7 per 1,000 person-years [17]. In the present study, it was estimated to be 116 per 1,000 person-years. This was higher than the incidence in the previous report because of several reasons. This figure included recurrence of psychosis in patients with a history of psychosis and clinic-based incidence, and anti-cholinergic drugs were used frequently. In the present study, the maximal length of the observation period was two years. Therefore, several factors such as cognitive function and PD severity could deteriorate during the observation period. In the survival analysis, patient-related factors were assumed to be stable because of limitations of the statistical methods. Psychosis is caused by multiple factors including patient-related factors and trigger medications, and there is no way to clearly differentiate psychosis due to drugs from that due to other conditions. Although these issues are limitations of the study, the results demonstrated that the severity of PD, cognitive dysfunction, and use of anti-cholinergic drugs were significant risk factors for psychosis. This result is consistent with those of previous studies [5,15-17,25]. In addition, sleep disturbances [25,29] and rapid eye movement (REM)-sleep behavioral disturbances [17] are associated with psychosis. Arousal systems, including nor-adrenaline neurons in the locus coeruleus, serotonin neurons in the raphe nuclei, and cholinergic neurons in the basal forebrain are damaged in PD [18,30]. Taken together, this information suggests that psychosis may be related to degeneration of cholinergic or serotonergic neurons. The use of anticholinergic drugs elevated the risk of psychosis and the use of donepezil reduced the risk, based on the results of **Analysis II**. This result is consistent with a previous report showing that rivastigmine, another acetylcholine esterase inhibitor, improves psychosis in PD [31] and these data suggest that psychosis is caused mainly by degeneration of cholinergic neurons in the brain arousal system. The function of brain cholinergic neurons is more severely affected in PD than in Alzheimer’s disease [32] and is impaired even in early stage of PD [33]. There was a significant interaction between PD duration and the use of anti-cholinergics because anti-cholinergics were previously used more frequently, and so were prescribed to patients with longer PD durations (data not shown). Although the mechanisms by which impairment of cholinergic neurons leads to psychosis are still unclear, the possibility that impairment of the inhibitory cholinergic circuit in the cerebral motor cortex is associated with visual hallucinations has been proposed [34]. Although psychosis might also be associated with sleep disorders, concomitant diseases, drugs other than those used in PD, hospitalization, or ocular disorders, these factors were not investigated in the present study.

Although no association between dopamine agonists and psychosis was identified in **Analysis II**, the risk posed by dopamine agonists was identified as being significant in the subgroup analysis of elderly patients. Relative risk of psychosis owing to dopamine agonists was 1.65 (1.02-2.66) per 100 mg (LDED) /day in patients aged ≥ 70 years, but it was not significant in patients aged <70 years. To clarify the risk owing to dopamine agonists in elderly patients, dose at the time of study enrollment in patients stratified by age (<70 versus ≥70) was examined. There was no significant difference in these doses between patients with psychosis and those without psychosis (Additional file 3: Table S1). These data show that dose escalation of dopaminergic drugs is a significant trigger in elderly patients but not in the entire cohort of patients, suggesting that dopamine agonists elevated the risk in psychosis-prone elderly patients but not in low-risk younger patients. In this context, psychosis could be avoided by careful application of dopamine replacement therapy in elderly or high-risk patients.

Because mild psychosis is not detected if patients or their caregivers do not complain of it, it is important how to define the occurrence of psychosis. In this study, the endpoint was defined as prescription of anti-psychotics. Neurologists intend to avoid prescribing anti-psychotics in patients with PD; therefore, the definition is a hard endpoint that has clinical significance. The participants in this study included patients with advanced disease and a history of psychosis. Therefore, the assessment of risk owing to medications estimated in this study cannot be extended to subjects with early-stage disease. In addition, it has not been elucidated whether there is a dose threshold for dopamine agonists to cause psychosis. To resolve these issues further examinations are required.

## Conclusions

The present study showed that psychosis is associated with the severity and duration of PD, and that patients with cognitive decline are prone to psychosis. After adjustment for these factors, anticholinergic drugs elevated the risk of psychosis, and in psychosis-prone elderly patients, dose escalation of dopamine agonists also elevated the risk.

## Abbreviations

PD: Parkinson disease; CI: Confidence interval; HR: Hazard ratio; mH-Y: Modified Hoehn-Yahr; MMSE: Mini-Mental State Examination; LDED: L-Dopa equivalent dose.

## Competing interests

This work was supported by a Clinical Research Grant from the National Hospital Organization (Dr Sawada).

Dr Sawada is funded by Grants-in-Aid from the National Hospital Organization and has received honoraria for lectures from GlaxoSmithKline and Boehringer Ingelheim. Dr Oeda is funded by Grants-in-Aid from the National Hospital Organization and has received honoraria for lectures from GlaxoSmithKline. Drs Umemura, Tomita, Yamamoto, Hayashi and Kawamura report no disclosures.

## Authors’ contributions

HS drafted the manuscript, had full access to all of the data in the study and takes responsibility for the integrity of the data and the accuracy of the data analyses. HS and TO provided the study concept and design. HS, TO, KY, AU, ST, and RH participated in the acquisition of data and analyzed the data. TK participated in critical revision of the manuscript. HS, TO, and TK performed the statistical analyses. All authors read and approved the final manuscript.

## Pre-publication history

The pre-publication history for this paper can be accessed here:

http://www.biomedcentral.com/1471-2377/13/145/prepub

## Supplementary Material

Additional file 1: Figure S1 Schematic demonstration of hazard and control periods. The red period represents the hazard period, in other words, the period with trigger medications. Medications were prescribed every 14 or 28 days; therefore, the duration of the hazard period was 14─28 days. Drugs taken in the hazard period were not changed in the period. Therefore, drugs that were taken 1 day before the endpoint (red arrow) represent the drugs prescribed in the hazard period. Similarly drugs that were taken 30 days or 90 days before the endpoint (blue period) represent drugs prescribed in the two control periods (blue arrows). Click here for file

Additional file 2: Figure S2 Correlation of medication doses at various time points (1, 30, and 90 days before the occurrence of psychosis). To confirm the assumption that the correlation of medication dose depends on the intervals between data collecting time points, doses of L-Dopa (A), dopamine agonists (B), selegiline (C), and amantadine (D) were plotted between 1, 30, and 90 days before the occurrence of psychosis. There were correlations between time points, and the correlation depended on the intervals between data collection time points. Therefore, data were analyzed using an autoregressive working correlation matrix as described in the Methods. Click here for file

Additional file 3: Table S1 Baseline dose of dopamine agonists (LDED mg/day) of patients who developed serious psychosis and those who did not, stratified by age. Click here for file
