# Supplementary material for: Trigger medications and patient-related risk factors for Parkinson disease psychosis requiring anti-psychotic drugs: a retrospective cohort study
Source: BMC Neurol. 2013 Oct 12;13:145. doi: 10.1186/1471-2377-13-145 (PMC3879653; doi:10.1186/1471-2377-13-145)
Supplement: Additional file 1: Figure S1 — Schematic demonstration of hazard and control periods. The red period represents the hazard period, in other words, the period with trigger medications. Medications were prescribed every 14 or 28 days; therefore, the duration of the hazard period was 14─28 days. Drugs taken in the hazard period were not changed in the period. Therefore, drugs that were taken 1 day before the endpoint (red arrow) represent the drugs prescribed in the hazard period. Similarly drugs that were taken 30 days or 90 days before the endpoint (blue period) represent drugs prescribed in the two control periods (blue arrows). [file 1471-2377-13-145-S1.pptx]

## Slide 1
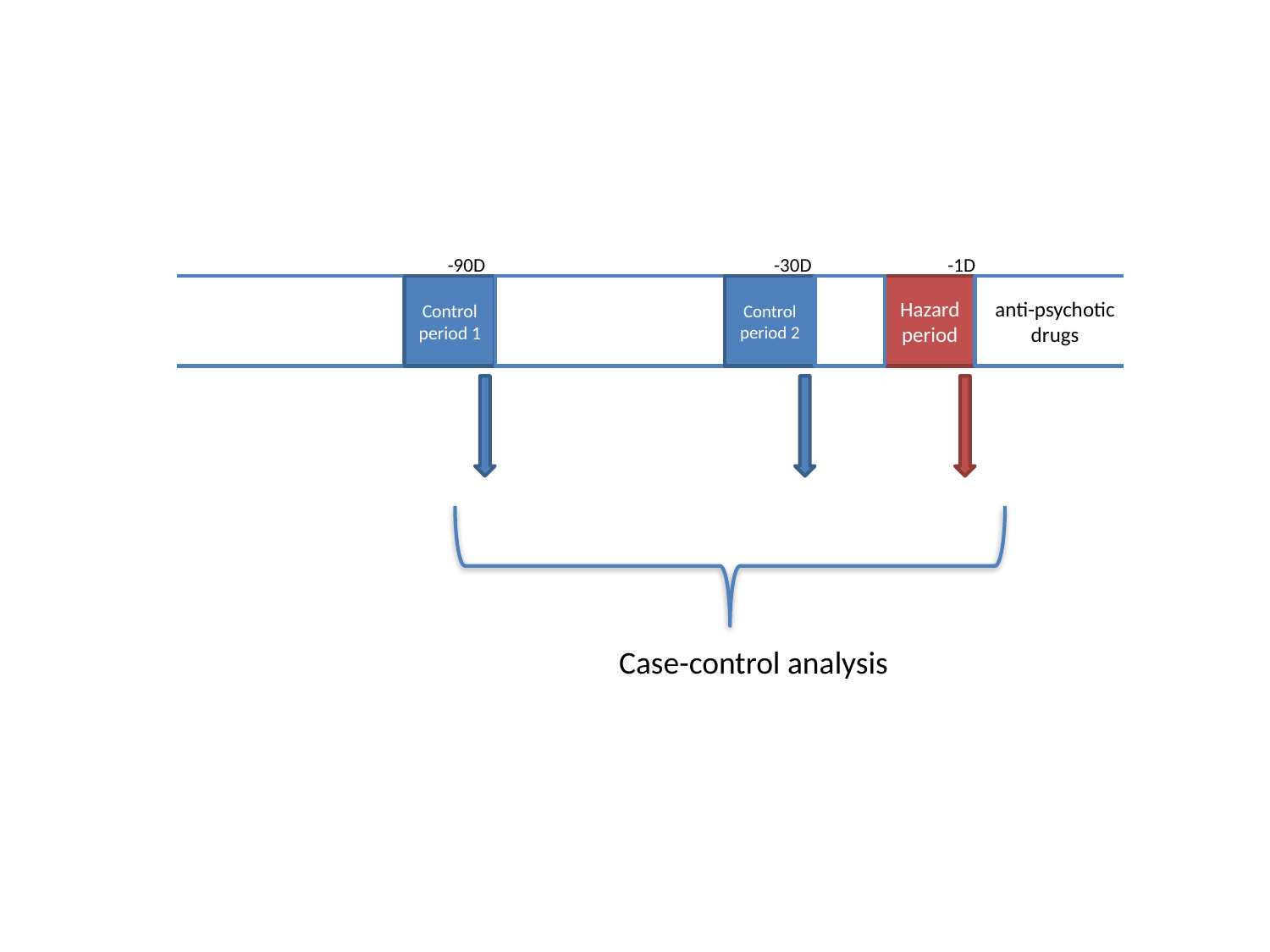

-90D
-30D
-1D
Control period 1
Control period 2
Hazard period
anti-psychotic drugs
Case-control analysis
