# Supplementary figures and images for: Trigger medications and patient-related risk factors for Parkinson disease psychosis requiring anti-psychotic drugs: a retrospective cohort study
Source: BMC Neurol. 2013 Oct 12;13:145. doi: 10.1186/1471-2377-13-145 (PMC3879653; doi:10.1186/1471-2377-13-145)

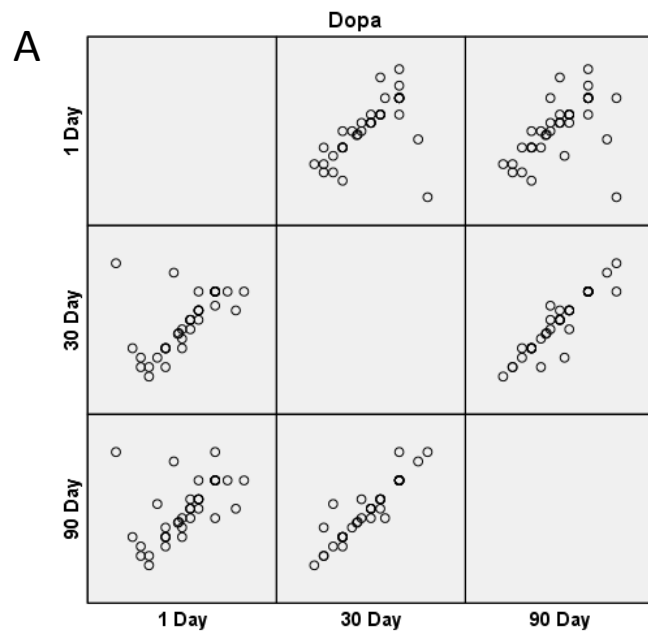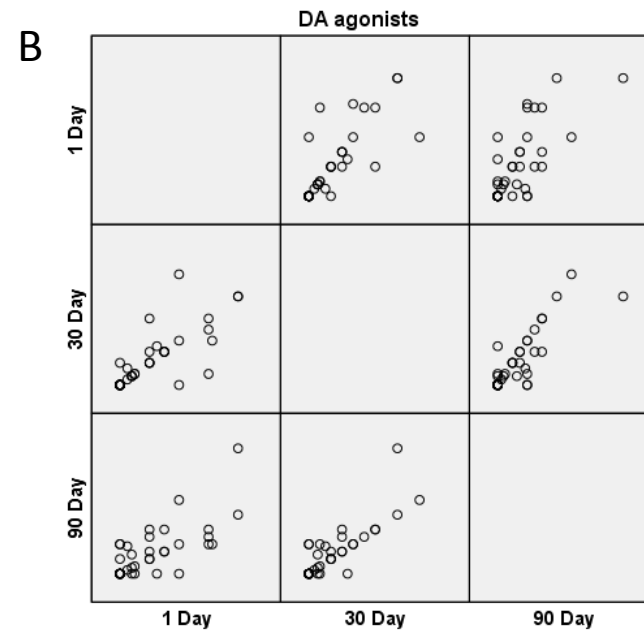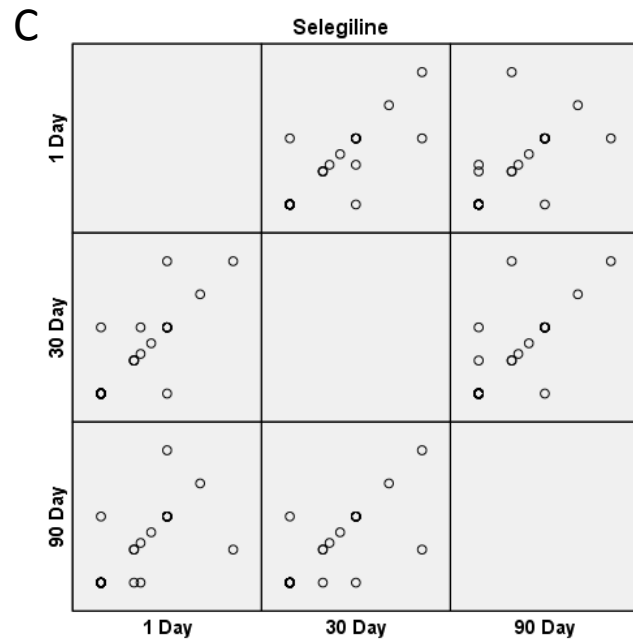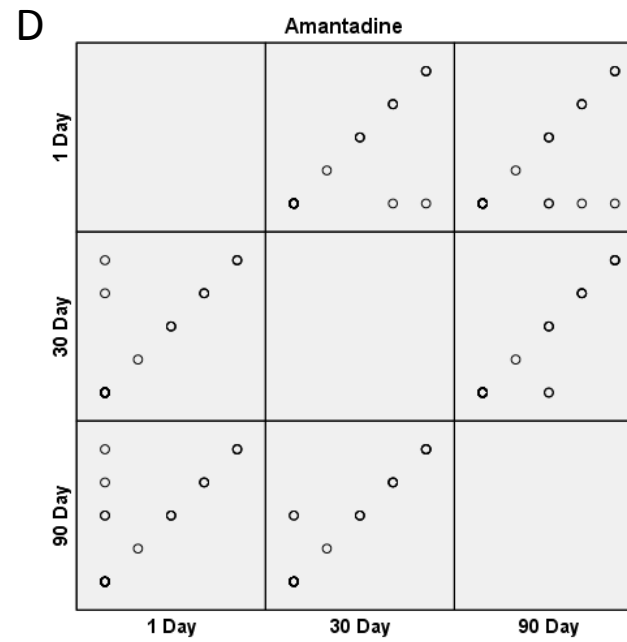

Supplement: Additional file 2: Figure S2 — Correlation of medication doses at various time points (1, 30, and 90 days before the occurrence of psychosis). To confirm the assumption that the correlation of medication dose depends on the intervals between data collecting time points, doses of L-Dopa (A), dopamine agonists (B), selegiline (C), and amantadine (D) were plotted between 1, 30, and 90 days before the occurrence of psychosis. There were correlations between time points, and the correlation depended on the intervals between data collection time points. Therefore, data were analyzed using an autoregressive working correlation matrix as described in the Methods. [file 1471-2377-13-145-S2.pdf]
